# Leveraging internet search data to assess prevalence, interest, and unmet needs of sarcoidosis in Germany

**DOI:** 10.1038/s41598-021-00131-x

**Published:** 2021-10-21

**Authors:** Charlotte Hilker, Linda Tizek, Melvin Rüth, Maximilian Schielein, Tilo Biedermann, Alexander Zink

**Affiliations:** 1 Department of Dermatology and Allergy, Technical University of Munich, School of Medicine, Biedersteiner str. 29, 80802 Munich, Germany; 2 Institute for Medical Information Processing, Biometry, and Epidemiology, Ludwig-Maximilians-University Munich, Munich, Germany; 3 Unit of Dermatology and Venerology, Department of Medicine, Karolinska Institutet, Karolinska University Hospital, Stockholm, Sweden; 4 Division of Dermatology and Venereology, Department of Medicine Solna, Karolinska Institutet, K2 Medicin, Solna, K2, 171 77 Stockholm, Sweden

**Keywords:** Skin diseases, Epidemiology, Outcomes research, Data acquisition

## Abstract

Sarcoidosis is a multisystemic disease of connective tissue with granuloma formation of unknown etiology and unclear prevalence. Internet search data has been shown to correlate with disease incidences and the population’s interest as well as seasonal variations. Accordingly, aim of this study was to leverage internet search data on sarcoidosis-related keywords to identify unmet needs, geographical and seasonal factors influencing sarcoidosis and estimating its prevalence. In this retrospective longitudinal study, Google Ads Keyword Planner was used to determine the internet search volume of terms related to sarcoidosis across Germany as a whole and in 17 major German cities between July 2015 and June 2019. Identified keywords were qualitatively categorized, converted into number of searches per 100,000 inhabitants and analyzed including regional and seasonal differences. With 3,068,200 queries and 425 different sarcoidosis-related search terms in the studied time period, the search volume was very high for a rare disease. Most searches (67.9%) related to general disease information with “sarcoidosis”, "Löfgren's syndrome", "sarcoidosis lung", "Morbus Boeck" and "neurosarcoidosis" as the top five keywords. Searches per 100,000 inhabitants were comparable in all 17 cities but higher than in Germany as a whole. Overall, the search volume increased from 2015 to 2019 and peaked annually in European springtime with annual lows in European autumn and winter months. The overall high search volume suggests an unmet need for sarcoidosis-related information and a diagnostic gap. Seasonal fluctuations indicate environmental as well as climatic factors that may influence sarcoidosis.

## Introduction

Sarcoidosis, also known as Boeck’s disease, is a multisystemic disease of connective tissue with granuloma formation and is described as the chameleon among the multisystemic diseases because of its varying manifestations, initial clinical signs, and course from patient to patient^1,2^. Histologically, non-necrotic granulomas with giant multinucleated cells dominate^1^. With an average lung involvement of about 90%, sarcoidosis most frequently causes pulmonary manifestations and often affects the mediastinal and hilar lymph nodes^3,4^. The prognosis depends on the type of sarcoidosis, the response to therapy, and the general condition of the individual^5^. Prevalence and incidence are not known for certain partly due to the disease’s nonspecific symptoms and its subsequent long diagnostic process^6,7^. In Europe, the annual sarcoidosis incidence is estimated at 5–60 cases per 100,000 inhabitants with a lifetime prevalence of 44 cases per 100,000 inhabitants^8,9^. Overall, it is estimated that there are about 30,000 cases in Germany with its total population of 83 million. The etiology of sarcoidosis is still unclear^5^. Currently, it is assumed that in a genetically predisposed person an immune dysfunction can be triggered by an as yet unidentified environmental factor^4^. This immune disorder, in turn, triggers a cascade which ends in a systemic granulomatous inflammation^5^. Infectious agents are repeatedly discussed as being possible environmental triggers, although a case–control study based on Swedish population registers found no significant correlation between infectious diseases and the development of sarcoidosis^10^. However, other studies concluded that exposure to aerosol infectious as well as seasonal and geographical variations in disease incidence could provide indications of the etiological significance of infectious agents^11–14^. Studies have also shown that the number of sarcoidosis cases varies within a country depending on the region. Although no clear urban or rural differentiation can be identified^15,16^.

There are no clear epidemiological data on prevalence and incidence of sarcoidosis in Germany. One approach to better understand the incidence and prevalence of diseases is to analyse the search volume of related search terms in online search engines such as Google, as the Internet is used as a common source of health information^17,18^. Approximately 95% of Germans use Google as a search engine in everyday life, which is why data tracking offers a variety of possibilities^19^. As demonstrated in previous studies, the analysis of Google search data is an unconventional approach to investigating people's needs and interest in various diseases^20,21^. Furthermore, websearch data has been shown to significantly correlate with the incidence of infectious diseases^22^. Hence, the aim of this study was to analyse nationwide and regional internet search data on sarcoidosis in Germany to identify unmet needs and geographical factors influencing the disease.

## Methods

### Study design

In this retrospective longitudinal analysis, Google Ads Keyword Planner was used to assess the search volume of terms related to the German word for sarcoidosis (‘Sarkoidose’) across Germany as a whole and in 17 large German cities (Berlin, Bremen, Cologne, Dresden, Erfurt, Frankfurt, Freiburg, Hamburg, Hannover, Kaiserslautern, Kiel, Munich, Muenster, Nuremberg, Rostock, Saarbrucken, and Stuttgart) between July 2015 and June 2019. Cities were chosen not only for their size but also for their geographic distribution across the country. In order to provide a better overview, the selected cities were allocated according to their geographical location as north, east, south and west and then graphically displayed together accordingly. The Keyword Planner is normally used in advertising to optimize marketing campaigns, but since the tool provides the monthly search volume of a specific topic, it can also be used for scientific purposes^23^. The term *search volume* applies to the number of searches for a topic or search term. To assess its volume within a specific field, words are initially entered into the Keyword Planner; thereupon, the program provides keywords that are most relevant to the topic. Only data from German IP addresses using Google in the German language were considered. Due to the described seasonal differences in sarcoidosis incidence, we defined the summer/spring months as March to August and the autumn/winter months as September to February^13^.

### Statistical analysis

**Figure 1:**
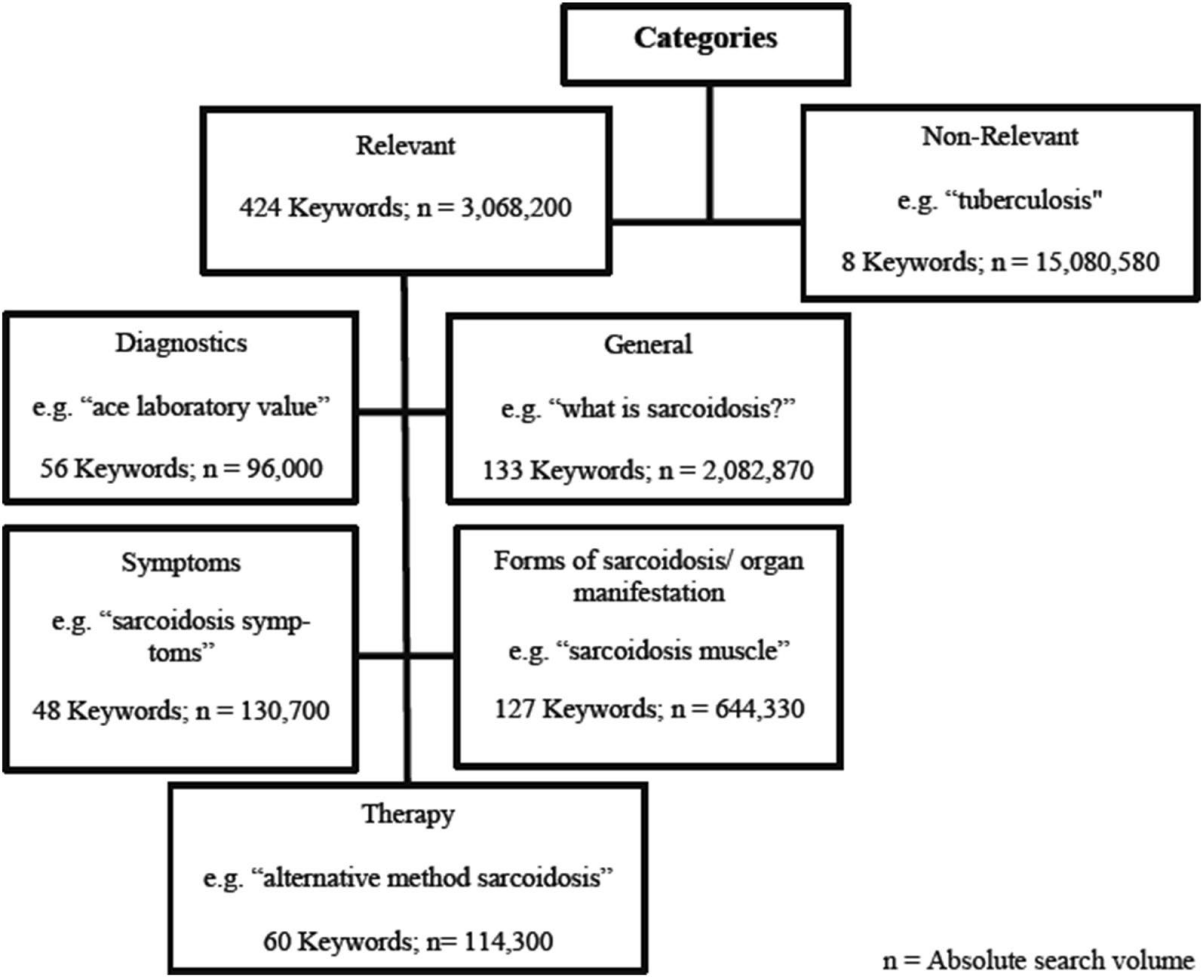
Categorization of the content of search terms identified by the Google Ads Keyword Planner.

All identified keywords were qualitatively assessed and classified by the authors. Keywords that were not relevant to sarcoidosis were excluded from analysis (e.g. “tuberculosis" "rheumatism"; Fig. 1), whereas search terms that did not directly contain the term sarcoidosis (e.g. “erythema nodosum” or “lung node”) but were medically associated with sarcoidosis were considered. All remaining keywords were further divided into five categories: “symptoms” (e.g. “sarcoidosis symptoms”), “diagnostics” (e.g. “ace laboratory value”), “form of sarcoidosis/organ infestation” (e.g. “sarcoidosis muscle”, “sarcoidosis nerves”, and “sarcoidosis lung”), and “therapy” (e.g. “alternative method sarcoidosis”). Search terms that did not fit into any of these categories were assigned to a “general” category (e.g. “sarcoidosis”, “Boeck’s disease”, and “what is sarcoidosis?”). Each search term was assigned to only one category. To compare differences in search behavior across Germany, the search volume of each city was divided by the respective number of inhabitants and then expressed per 100,000 inhabitants. Geodata for administrative boundaries from the German Federal Agency for Cartography and Geodesy were collected using the geographic information system QGIS, version 2.14.22 (QGIS Development Team)^24^.

## Results

Overall, 433 keywords associated with sarcoidosis were identified using the Keyword Planner. Of those, eight keywords were excluded from the final analysis as they were not relevant. The remaining 424 keywords had an overall search volume of 3,068,200 queries across Germany from July 2015 to June 2019 (Fig. 1). The category "general" had the highest number of search queries with 2516 queries per 100,000 inhabitants (67.9%). Within this category, the terms “sarcoidosis”, “Boeck's disease”, “systemic disease”, “sarcoidosis nutrition” and “sarcoidosis lung life expectancy” (in descending order), represented the most searched terms. The second most searched category was "form of sarcoidosis/organ manifestation" with 778 queries per 100,000 inhabitants (21.0%). When considering the localization of sarcoidosis, people searched for almost all organ systems. With 19.8% of all keywords, people searched most frequently for the lung with keywords like "ace lung", "autoimmune disease lung", and "granulomatous lung disease". The five most commonly searched keywords were “sarcoidosis” (2,041 searches/100,000 inhabitants), "Löfgren's syndrome" (120 searches/100,000 inhabitants), "sarcoidosis lung" (99 searches/100,000 inhabitants), "Morbus Boeck" (91 searches/100,000 inhabitants), and "neurosarcoidosis" (69 searches/100,000 inhabitants, Tables 1 and 2).

**Table 1:** The most frequently searched keywords related to sarcoidosis in Germany. The figure contains percentages related to 100,000 inhabitants and the percentage share of the search term in the total search volume.

Keyword	Category	Search volume per 100,000 inhabitants	Percentage of the total search volume
Sarcoidosis	General	2040.5	55.1
Löfgren’s syndrome	Form of sarcoidosis/organ manifestation	120.0	3.2
Sarcoidosis lung	Form of sarcoidosis/organ manifestation	98.9	2.7
Morbus Boeck	General	90.6	2.4
Neurosarcoidosis	Form of sarcoidosis/organ manifestation	68.5	1.8
Sarcoidosis skin	Form of sarcoidosis/organ manifestation	50.3	1.4
Sarcoidosis symptoms	Symptoms	43.0	1.2
Pulmonary sarcoidosis	Form of sarcoidosis/organ manifestation	31.4	0.8

**Table 2:** The five most frequently searched keywords related to sarcoidosis per category in the whole of Germany.

Category	Keywords	Absolute search volume	Search volume per 100,000 inhabitants
General	Sarcoidosis	1,689,300	2040.5
	Morbus Boeck	75,000	90.6
	Systemic disease	20,100	24.3
	Sarcoidosis nutrition	19,200	23.2
	Sarcoidosis lung life expectancy	18,730	22.6
Form of sarcoidosis/organ manifestation	Löfgren’s syndrome	99,380	120.0
	Sarcoidosis lung	81,910	98.9
	Neurosarcoidosis	56,740	68.5
	Sarcoidosis skin	41,650	50.3
	Lungsarcoidosis	25,980	31.4
Symptoms	Sarcoidosis symptoms	35,610	43.0
	Pulmonary hyperinflation	20,150	24.3
	Sarcoidosis skin pictures	9340	11.3
	Sarcoidosis skin itching	7120	8.6
	Neurosarcoidosis symptoms	6500	7.9
Therapy	Sarcoidosis therapy	23,880	28.8
	Sarcoidosis forum	18,730	22.6
	Sarcoidosis specialist	5600	6.8
	Sarcoidosis treatment	4180	5.0
	Sarcoidosis therapy guidelines	3350	4.0
Diagnostics	Sarcoidosis diagnostics	16,250	19.6
	Sarcoidosis values	6370	7.7
	Sarcoidosis laboratory	5580	6.7
	Ace sarcoidosis	4690	5.7
	Sarcoidosis X-ray	3580	4.3

### Comparisons between cities

**Figure 2:**
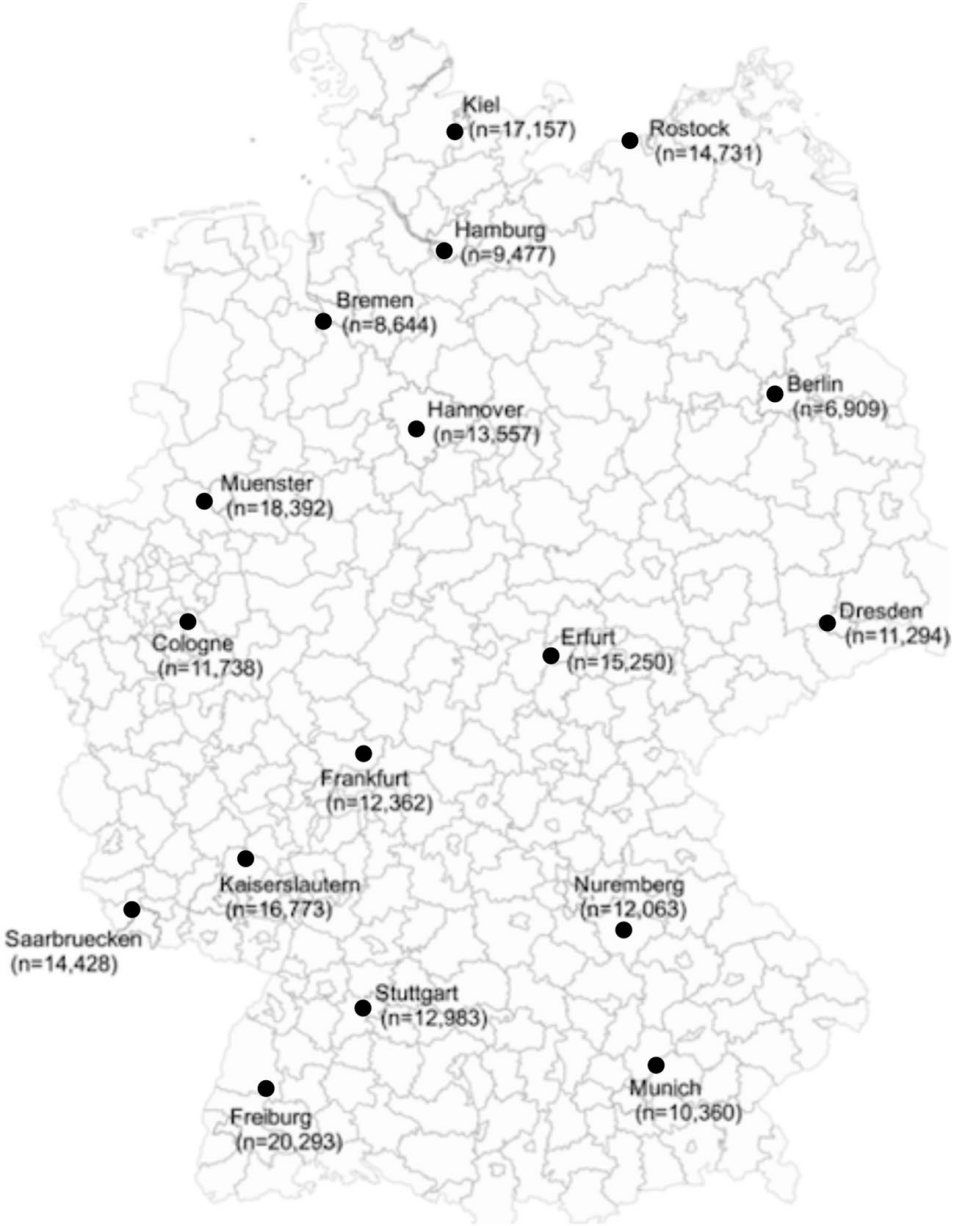
Search volume per 100,000 inhabitants of terms related to sarcoidosis in 17 large German cities between July 2015 and June 2019. QGIS.org, 2021. QGIS Geographic Information System. QGIS Association. http://www.qgis.org.

Differences in search frequency were identified between cities, but no considerable differences in search frequency were observed within the geographical regions. In all 17 cities examined, the number of search queries per 100,000 inhabitants was higher than the national average (3706 searches/100,000 inhabitants). The cities with the lowest relative search volume were Berlin (6909 searches/100,000 inhabitants), Bremen (8644 searches/100,000 inhabitants), and Hamburg (9677 searches/100,000 inhabitants). In contrast, the highest number of searches per 100,000 inhabitants was observed in Freiburg (20,293 searches/100,000 inhabitants), followed by Muenster (18,392 searches/100,000 inhabitants) and Kiel (17,157 searches/100,000 inhabitants, Fig. 2). Berlin, with a population of about 3.6 million inhabitants (3,613,495), had a lower search volume per 100,000 inhabitants than Kaiserslautern, with a population of just under 100,000 (99,864). The same can be observed in Hamburg (n = 9477, inhabitants = 1,830,584) and Freiburg (n = 20,293, inhabitants = 229,636), as well as in Munich, Kiel and Rostock (Fig. 2). Looking at the categorical distribution (Table 3), a larger proportion of search queries in the cities fell into the categories of sarcoidosis forms/organ manifestation, diagnostics, symptoms, and therapy than into the general category. In Freiburg, for example, 11% of search queries occurred in the category “diagnostics” and 46% in the general category, whereas the national percentages for these categories were 3% and 68%, respectively. This discrepancy between city and national percentages can also be observed in Kaiserslautern, where 35% of the searches fell into the category “sarcoidosis forms/organ infestation” and in 41% into the category “general”, whereas the national percentage for the former category was 21%. The categorical distributions in Berlin and Hamburg were similar to the national distribution pattern (Table 3).

**Table 3:** Proportional distribution of the search volume according to categories of sarcoidosis-related keywords in Germany and 17 major German cities between July 2015 and June 2019.

	Number of inhabitants	Number of searches	Number of searches per 100,000 inhabitants	Internet searches percentage distribution according to the categories				
				Diagnostics (%)	General (%)	Form of sarcoidosis/organ manifestation (%)	Symptoms (%)	Therapy (%)
Germany	83,000,000	3,068,200	3706.0	3	68	21	4	4
Berlin	3,613,495	249,660	6909.1	7	**57**	24	**6**	**6**
Bremen	568,006	49,100	8644	8	47	30	7	8
Dresden	551,072	62,240	11,294	8	48	29	7	7
Erfurt	212,988	32,480	15,250	11	44	30	8	7
Frankfurt	746,878	92,330	12,362	9	50	27	7	7
Freiburg	229,636	46,600	20,293	11	*46*	*30*	*6*	*7*
Hamburg	1,830,584	173,490	9477	8	**54**	**25**	**7**	**7**
Hannover	535,061	72,540	13,557	9	49	28	6	7
Kaiserslautern	99,684	16,720	16,773	8	*41*	*35*	*8*	*8*
Kiel	247,943	42,540	17,157	9	47	30	7	7
Cologne	1,080,394	126,820	11,738	8	52	26	7	7
Munich	1,456,039	150,840	10,360	9	53	25	6	7
Muenster	313,559	57,670	18,329	11	46	30	6	8
Nuremberg	515,201	62,150	12,062	9	47	29	8	8
Rostock	208,409	30,700	14,730	9	45	32	7	7
Saarbruecken	180,966	26,110	14,428	10	*42*	*33*	*8*	*7*
Stuttgart	632,743	82,150	12,983	9	49	27	7	7

Diagnosis, general, form of sarcoidosis/organ manifestation, symptoms, therapy provide the names for the categories attributed to the search terms: “symptoms” (e.g. “sarcoidosis symptoms”), “diagnostics” (e.g. “ace laboratory value”), “form of sarcoidosis/organ infestation” (e.g. “sarcoidosis nerves”), “therapy” (e.g. “alternative method sarcoidosis”) and “general” category (e.g. “sarcoidosis”, “Boeck’s disease”). Bold similar to Germany-wide distribution, italics diverging distribution pattern compared to Germany-wide categorical distribution.

### Time course of search behavior

**Figure 3:**
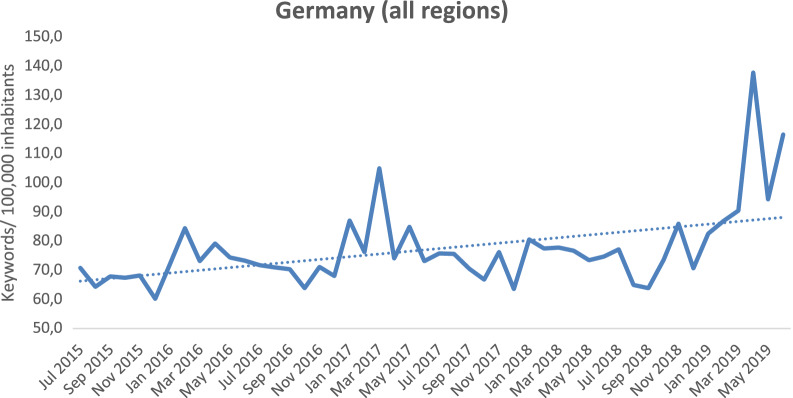
German national wide number of Google search queries related to sarcoidosis from June 2015 to July 2019 per 100,000 inhabitants (424 keywords; n = 3,068,200, nr = 3706.0 searches/100,000 inhabants).

The national search volume of Google search queries related to sarcoidosis increased from July 2015 to June 2019 for Germany (Fig. 3). The month with the lowest search volume per 100,000 inhabitants was December 2015 (60 searches/100,000 inhabitants), whereas April 2019 had the highest search volume (138 searches/100,000 inhabitants). Overall, the data indicated a small increase in search volume within the study period. Especially, in European springtime (March–May) people tended to search more often for sarcoidosis, whereas in European winter (December–February) a decrease in search volume was observed.

**Figure 4:**
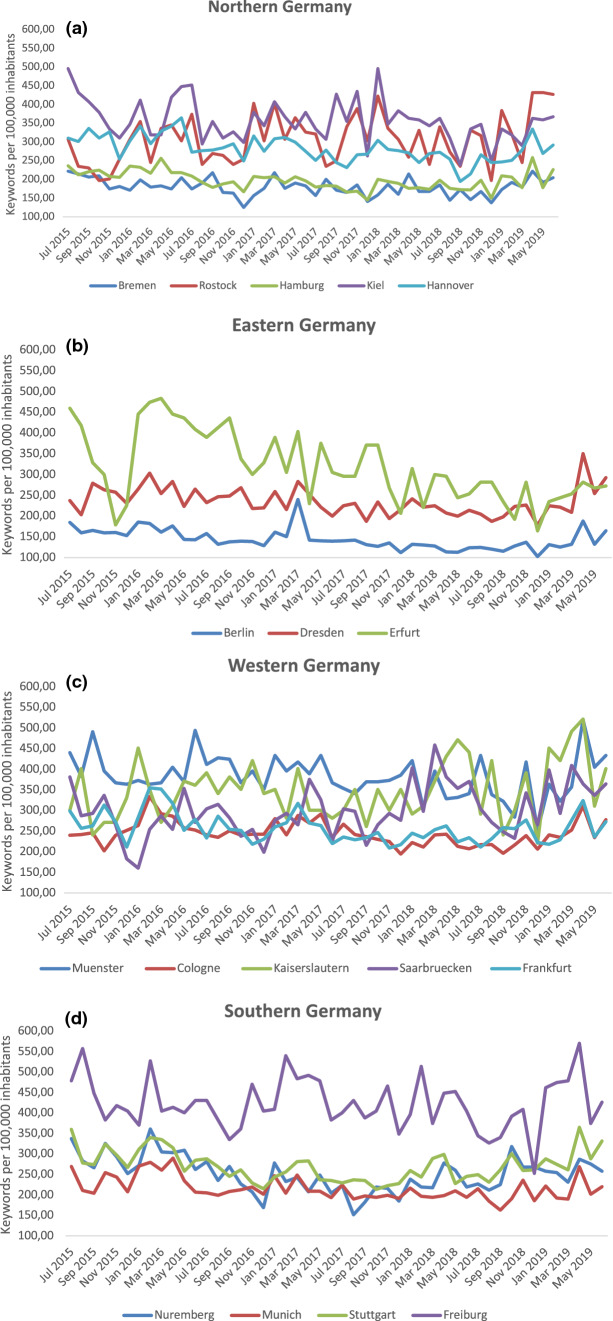
(**a**) Number of Google search queries on sarcoidosis in Northern Germany from June 2015 to July 2019 per 100,000 inhabitants. (**b**) Number of Google search queries on sarcoidosis in Eastern Germany from June 2015 to July 2019 per 100,000 inhabitants. (**c**) Number of Google search queries on sarcoidosis in Western Germany from June 2015 to July 2019 per 100,000 inhabitants. (**d**) Number of Google search queries on sarcoidosis in Southern Germany from June 2015 to July 2019 per 100,000 inhabitants.

Furthermore, the 17 cities were further divided based on their geography into groups of northern, southern, eastern, and western cities to get an overview of the monthly range in search behavior, based on geographical differences. In Northern Germany, Kiel had the highest and Bremen the lowest number of search queries (Fig. 4a). In Eastern Germany, the number of search queries in Erfurt was higher than that in Dresden and Berlin (Fig. 4b), with a particularly sharp increase from February to June 2016. A wide range in search volume was observed in cities in Western Germany, where for example the search volume ranged between 222 per 100,000 inhabitants (December 2018) and 522 per 100,000 inhabitants (April 2019) for Kaiserslautern. In Southern Germany, Freiburg had the highest volume during the whole study period, with high values in July 2015 (557 searches/100,000 inhabitants), and April 2019 (570 searches/100,000 inhabitants), which were the highest recorded search volumes among all cities throughout the entire study period. All cities recorded a distinct increase in April 2019 (Fig. 4a–d).

## Discussion

The aim of the study was to identify areas of unmet need and geographical as well as seasonal factors that influence sarcoidosis as the basis for optimizing sarcoidosis care by leveraging Internet search data. The most commonly searched was the word “sarcoidosis”, followed by more specific search terms such as “Löfgren's syndrome” and “sarcoidosis lung”. Furthermore, even more specific diagnostic terms such as “ACE laboratory value” were searched for. The search volume of these relatively specific keywords might imply that affected individuals search for information online using terms they have heard as part of their own diagnosis or heard from individuals who have received a sarcoidosis diagnosis.

The three most frequently searched terms, “sarcoidosis”, “Löfgren’s syndrome”, and “sarcoidosis lung”, are representative of their respective diagnoses, since sarcoidosis summarizes all sarcoidosis subtypes, Löfgren’s syndrome is found in up to 50% of all cases, and the lung is the most frequently affected organ^5^. This is further emphasized by the fact that two of the five most frequently searched keywords are organ manifestations ("sarcoidosis skin" and "sarcoidosis lung"), which reflect how this multisystemic disease often manifests itself first in the lungs and the skin^5,25^. The high interest in neurosarcoidosis seen with "neurosarcoidosis" being among the top five search terms is notable considering since it is a rare organ manifestation that only occurs in 5–10% of all cases in conventional studies^26^. This might underline people's fear of this sarcoidosis subtype or just an exaggerated interest due to the rarity of neurosarcoidosis. For example, “eye melanoma” is a rare disease shown to have similarly high interest in the population^21^. Another explanation is an increased interest stemming from individuals who show neurological symptoms but have not been diagnosed with neurosarcoidosis informing themselves online on this rare organ manifestation.

The study results indicate an increase in search volume for sarcoidosis during springtime. One study does indicate differences in terms of decreasing numbers in autumn compared to higher numbers in spring, which however, where not statistical significance^13^. One possible explanation for the observed increase in search volume in this study is the preceding wave of influenza. Granulomas of (symptomless) pulmonary sarcoidosis are often discovered by chance in chest X-rays, which are performed to identify influenza complications^27^. Another possible explanation is the strain on the immune system by high pollen counts in the spring that prompt inflammation of the respiratory epithelium through oxidase and neutrophil activity. This could augment the risk for developing sarcoidosis and exacerbate chronic sarcoidosis symptoms^28,29^. This assumption is supported by the fact that every year the volume of search terms was higher in April and May than in the other months. The deterioration of chronic lung diseases caused by pollen has already been observed in other studies on asthma^30,31^. Climate differences could also play a role, which would explain the decline in searches during the winter months. However as mentioned above, in other studies no statistically significant differences could be found, which makes the results of this websearch analysis even more interesting^13^. Furthermore, April 2019 was particularly striking with search results rising to 138 searches/100,000 inhabitants. That year the Foundation for Sarcoidosis Research (FSR) declared April “Sarcoidosis Month” and through campaigns sought to increase awareness about the disease, which would possibly explain this substantial peak in search volume.

### Keyword distribution in different German cities

The result showed that the search volume per 100,000 inhabitants was sustainable higher in smaller cities such as Freiburg and Muenster than in larger cities like Berlin and Hamburg. The lower number of search queries in bigger cities might be explained by the better access to medical care with specialists in larger cities than in smaller cities. There are some studies on the over- and undersupply of specialists in rural areas, such as neurologists and psychiatrists, but none specifically related to sarcoidosis^32,33^. However, it can be assumed that there are differences in access to specialists between urban and rural regions because of disparities in infrastructure and the allocation of health insurance fund seats according to the density of the patient population^34^. Previous studies have shown that people living in rural areas are less likely to visit specialists^35^. Accordingly, people living closer to specialists might consult a specialist instead of using Google for health-related information. In the case of sarcoidosis, this could mean that people living in rural areas are less likely to be diagnosed^19,36^. In smaller cities people might get the diagnosis, but as the medical care to specialists is less in comparison to big cities, they have less opportunity to ask questions about the disease. This deficit in information supply might be compensated by search queries. Another explanation could be that smaller cities like Freiburg, Muenster, Kiel, and Rostock, have a higher proportion of medical students and healthcare professionals within the respective inhabitants than large cities do and that the higher search volume of sarcoidosis related keywords in these cities can be attributed to medical students performing online research on the disease. Overall, the search queries in each city were higher than the national average. This was particularly noticeable in Kiel, Freiburg, and Erfurt. This phenomenon could also be explained by the lack of diagnosis in rural areas. The high search volume in Freiburg may be explained by the presence of the National Action Alliance for People with Rare Diseases, which has established appropriate structures for patient care in the University Hospitals of Freiburg and Tübingen^37^. Furthermore, it was shown that older people use the Internet less common, which might be one reason for the lower number of search queries in the whole of Germany compared to the cities examined^38^. Assuming that a higher search volume correlates with a higher prevalence of sarcoidosis like shown in studies on melanoma^36^, the study results contradict those from a previous study that found a higher prevalence among people living in rural areas^12^. The results of the Google analysis could accordingly indicate a much higher prevalence of sarcoidosis than previously assumed.

Ultimately, the discrepancy in search volume between individual cities and Germany as a whole can suggest an actual variation in sarcoidosis prevalence, but it also reflects different demographic distributions and better availability of resources, closer networking such as self-help groups and medical care in urban areas.

### Limitations

There are some study limitations. Google analyses are limited to people who have access to the Internet and use Google as a search engine. Although 95 percent of Germans use Google as a search engine, only including data collected from Google excludes information from a portion of the population. In general, young people use the Internet more than older people. On user demographics, the study is not able to show correlations with people’s age, gender, or nationality. Accordingly, the study might underestimate the disease burden through exclusion of elderly members of the population as well as foreign, non-German speaking residents. Another limitation of the present study is that the searches do not necessarily have to originate from affected individuals, but also from physicians, medical students, or relatives who are interested in the topic as well. It must be taken into consideration that most of the cities included in the study have large medical schools, which can lead to a disproportionately high number of searches through online student activity and medical research. Accordingly, this could be another explanation for the higher number of searches per 100,000 inhabitants in Freiburg for example. Furthermore, there is no information whether a few individuals searched very often or whether many people searched once or twice. So far, there is no study that assessed the correlation between online searches and the prevalence of sarcoidosis. However, considering other studies, a correlation between the number of Google searches and the number of registered cases such as skin cancer or multiple sclerosis was found^36,39^. Another study reported that online tools play an important role in gathering information regarding rare diseases^40^. Based on these findings, it can be assumed that there is a relation between search behavior and the occurrence of sarcoidosis. Another limitation is that Google through its autocomplete function provides search suggestions that could as well distort search behavior. Moreover, data provided by Google Ads Keyword Planner are based on monthly estimations instead of exact numbers and thus might slightly differ from the actual search volume. Therefore, the search behavior in cities might be rather overestimated, while it might be underestimated for Germany as a whole^19,36^.

## Conclusion

The study demonstrated that there is an unmet need for information within affected individuals and the population for sarcoidosis-related keywords, as the search volume for this rare disease is relatively high in relation to the estimated prevalence in Germany. Another perspective of course could be that the prevalence is higher than previously assumed based on conventional studies, given the high number of searches in the cities in comparison to the overall search volume across the whole of Germany. Furthermore, low web search numbers in rural areas indicate a diagnostic gap and the clearly increased web search frequency in spring suggests environmental and climatic trigger factors, worthy of further investigation.

## Data Availability

All data generated or analysed during this study are included in this published article.
